# Coronary Artery Aneurysms as a Cause of Acute Coronary Syndrome Presentation - A Focused Review

**DOI:** 10.2174/1573403X19666230331103508

**Published:** 2023-07-17

**Authors:** Azka Latif, Amy Tran, Junaid Ahsan, Noman Lateef, Waiel Abusina, Vikas Kapoor, Zoraiz Ahsan, Soban Ahmad, Mohsin Mirza

**Affiliations:** 1 Division of Cardiovascular Medicine, Baylor College of Medicine, Houston, Texas, USA;; 2 Department of Medicine, Creighton University Medical Center, Omaha, Nebraska, USA;; 3 Division of Cardiovascular Medicine, Mercy Medical Center, Iowa Heart Center, Des Moines, Iowa, USA;; 4 Division of Cardiovascular Medicine, University of Nebraska Medicine, Omaha, Nebraska, USA;; 5 Department of Medicine, CHI Health Good Samaritan Hospital, Kearney, Nebraska, USA;; 6 Department of Medicine, Pakistan Medical Center, Islamabad, Pakistan;; 7 Department of Medicine, East Carolina University/Vidant Medical Center, Greenville, North Carolina

**Keywords:** Coronary artery aneurysms, acute coronary syndrome, coronary artery disease, complications, management, CAAR

## Abstract

Coronary artery aneurysms (CAA) are defined as a dilation of a coronary vessel greater than 1.5 times the diameter of a local reference vessel. While CAAs tend to be incidental findings on imaging, they result in complications, such as thrombosis, embolization, ischemia, arrhythmias, and heart failure. Among symptomatic cases, chest pain has been the most common manifestation of CAAs. This necessitates an understanding of CAAs as a cause of acute coronary syndrome (ACS) presentation. However, due to the unclear pathophysiology of CAAs and their variable presentation complicated by similar ACS conditions, there is no clear strategy for CAA management. In this article, we will discuss the contribution of CAAs to ACS presentations and review the current management options for CAAs.

## 1 INTRODUCTION

According to the international Cardiac Artery Aneurysm Registry (CAAR), the prevalence of CAAs is estimated to be 0.35-4.9% among individuals undergoing cardiac catheterization procedures. With the widespread use of investigational studies, it is expected that there will be an increase in the discovery of patients with CAAs [1]. CAAs most commonly affect the left anterior descending artery and right coronary artery followed by the circumflex artery [1, 2]. Currently, CAAs can be classified by the following categories: true aneurysm versus pseudoaneurysm, appearance, size, and extent of involvement of the coronary arteries. First, the distinction between true aneurysms and pseudoaneurysms depends on the number of involved vessel layers. True aneurysms involve all three vessel layers: tunica intima, media, and adventitia. In contrast, the disrupted exterior elastic membrane in pseudoaneurysms results in a loss of wall integrity and subsequent involvement of only one or two layers of the vessel wall [3]. Second, there are two sub-classifications for CAA morphology: saccular and fusiform. Saccular forms have a transverse diameter greater than the longitudinal diameter, while fusiform types have a longitudinal diameter greater than the transverse diameter. Third, the size of CAAs has been defined as a dilation of a coronary vessel greater than 1.5 times the diameter of a local reference vessel. Some reports have described giant CAAs, which are defined as dilations exceeding 4 times the diameter of a local reference vessel or 50 mm in adults and 8 mm in pediatric populations, although the exact size criteria continue to be under debate [4, 5]. Lastly, CAAs can be classified into four types based on their extent of involvement: diffuse dilation of two or three vessels (Type I), diffuse dilation in one vessel and localized disease in another (Type II), diffuse dilation of one vessel only (Type III), and localized or segmental dilation (Type IV) [5].

Current imaging modalities to evaluate suspected CAAs include coronary angiography (CAG), coronary magnetic resonance angiogram (MRA), coronary computed tomography angiogram (CTA), intravascular ultrasound (IVUS), and echocardiography. Although invasive, CAG remains the most widely used and the gold standard for diagnosing CAAs as it provides information about morphology, size, location, and potential complications, such as myocardial perfusion abnormalities, and insights for a surgical resection approach. However, the true size of CAAs may be underestimated if there is a significant thrombus or plaque burden [5, 6]. As an alternative to CAG, CTA can be recommended for CAA follow-up due to its non-invasive modality and more accurate assessment of aneurysm size, thrombus, and calcification as compared to CAG [5, 6]. Echocardiography and MRA do not involve high-dose radiation, unlike CAG or CTA. However, these methods better characterize CAAs in larger proximal coronary artery segments rather than smaller distal segments [5, 6]. As another non-invasive imaging modality, IVUS provides transmural pictures of coronary arteries, including information about arterial walls and lumen anatomy. Thus, IVUS is highly beneficial for differentiating a true aneurysm from a pseudoaneurysm in the setting of plaque rupture [5, 6]. Each imaging modality offers its own benefits and drawbacks to characterizing CAAs. Therefore, effective evaluation of CAAs often requires multimodality imaging [6].

Although the exact pathophysiology of CAAs has not been yet identified, atherosclerosis is commonly considered to be the primary cause of CAAs among adults [7, 8]. Additional predisposing characteristics include male gender and cardiovascular risk factors such as hypertension, dyslipidemia, diabetes mellitus, smoking, and obesity [7]. Histological examination of CAAs suggests that atherosclerosis can contribute to aneurysm formation through compromised structural integrity from disturbed intimal and medial layers of arterial walls, destroyed elastic muscular components, the presence of cholesterol crystals, and areas of calcification and fibrosis. These characteristics create weaker and more rigid vessel walls, which decreases tolerance to intraluminal pressures and predisposes to vascular dilation and aneurysm formation. In addition, the persistent transmural inflammation seen in atherosclerotic disease can exacerbate worsening vessel wall strength and subsequent aneurysm development [9, 10].

Other causes of CAAs include Kawasaki disease, vasculitis, and vessel wall injury following percutaneous coronary intervention (PCI) [8, 11]. The most well-known association for CAAs in young children is Kawasaki disease. Unlike the process of atherosclerosis in adults, the pathogenesis of CAAs in Kawasaki disease is postulated to be related to the active remodeling process of coronary arteries due to vascular growth factors [12]. Necrotizing vasculitis in Kawasaki disease is accompanied by neutrophilic infiltration, which damages the tunica intima, media, and some of the adventitia of coronary arteries. Recruitment of inflammatory cells leads to cytokine release, luminal myeloblast proliferation, and subsequent obstruction of the coronary lumen [13, 14].

## 2 ACUTE CORONARY SYNDROME SECONDARY TO CORONARY ARTERY ANEURYSMS

CAAs are often discovered as incidental findings in patients who undergo coronary angiography or computed tomography for other cardiovascular conditions. Among symptomatic cases of CAAs, exertional angina is the most frequent presentation [5]. Some studies suggest CAAs as a variant of coronary artery disease (CAD), accounting for half of the documented cases. Swayne *et al.* found no significant differences between aneurysmal and non-aneurysmal coronary disease patients with regard to atherosclerotic cardiovascular risk. They concluded that aneurysmal CAD is not likely a distinct clinical entity but rather a variant of CAD [11]. Thus, CAAs can manifest as subacute or acute presentations of ACS, which makes their diagnosis more challenging. Local thrombosis, distant embolization, or aneurysmal rupture can contribute to ACS and result in severe complications. Although atherosclerosis is thought to be the main contributing factor to CAAs, several cases have reported that local CAA thrombosis can result in acute myocardial infarction (MI) even without any significant past cardiovascular history [15-17]. One proposed explanation for the presence of anginal symptoms without obstructive lesions in CAA patients is coronary slow flow (CSF). This phenomenon results in a mismatch between coronary blood flow and oxygen demand, and thus increases the risk for stress-induced myocardial injury and ischemia. The underlying pathophysiology is postulated to be related to coronary endothelial dysfunction [18].

## 3 MANAGEMENT OF CAAS

**Fig. (1):**
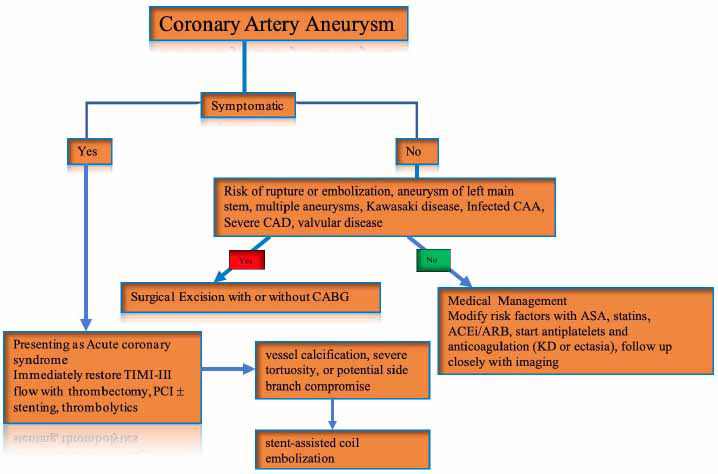
Management of coronary artery aneurysm.

Available management options for CAAs are medical therapy, PCI, and surgical procedures. Management strategies are tailored to individuals based on patient characteristics, clinical presentation, underlying etiology, and CAA features (Fig. **1**). In general, asymptomatic patients without a history of CAD do not require any treatment. Among those with symptomatic CAAs, there is no clear standardized treatment approach, which necessitates further future investigations in this area [8].

### 3.1 Medical Therapy

Atherosclerosis is a common underlying cause of CAAs as it is responsible for more than 50% of CAAs in adults, and thus, aggressive risk factor modification is critical for the reduction of complications and improvement of prognosis [6]. However, there are no clear guidelines for the management of CAAs, which presents a therapeutic challenge to physicians, especially interventional cardiologists [7]. In patients with CAAs due to a thrombotic etiology or those who have undergone PCI, the goal is to reduce the risk of future thrombosis. The role of dual antiplatelet therapy (DAPT) and warfarin in patients with CAA is still a topic of debate. The recommended regimen is aspirin, which is the preferred choice as a single medicine or along with another anticoagulant or antiplatelet agent depending on clinical circumstances [8, 6]. CAAs account for 2-3% of PCI complications [19]. Specifically, a 12-month course of DAPT following stent placement is strongly suggested to decrease the risk of thrombus formation [20]. On the other hand, a retrospective analysis by Khubber *et al.* showed DAPT and anticoagulation to not be associated with significant benefits in the outcomes of CAAs treatment [21]. Also, Doi *et al.* found that patients who were on anticoagulation therapy in the target therapeutic range of ≥ 60% did not experience any major adverse cardiac event (MACE) compared to patients maintained in a sub-optimal therapeutic range [22]. Other medical management options include angiotensin convertase enzyme (ACE) inhibitors and statins to prevent the progression of CAAs, although there is no definitive evidence [6].

### 3.2 Percutaneous Coronary Intervention

PCI is indicated for CAA patients who present with ischemia or acute STEMI where the goal is to restore blood flow [7, 8]. Compared to other ACS conditions, PCI for CAAs has less success and a higher risk of re-embolization, repeat thrombosis, and long-term mortality [7]. These adverse effects are due to the technical challenges of PCI in managing CAAs, such as difficulties in delivering covered stents to their target site. These stiff stents can be deployed incorrectly in settings of vessel calcification, severe tortuosity, or potential side branch compromise. In such cases, stent-assisted coil embolization is the preferred method for CAA management [7]. Thus, the choice of the PCI method is highly dependent on the CAA architecture.

Although bare-metal stents (BMS) and drug-eluting stents (DES) have similar long-term clinical outcomes, first- and second-generation DES are preferred due to their lower rates of in-stent restenosis (ISR) and late thrombosis [23, 24]. CAAs are rare complications associated with PCI [25]. The true rate of CAA after PCI is still largely unknown. The reported incidence of CAAs after induction of DES is 0.2%-2.3%; similar rates have been reported after the induction of bare-metal stents, accounting for 0.03%-3.9% [19]. In their study, Hong *et al.* discovered no significant difference in CAA incidence between first- and second-generation DES. However, CAAs after second-generation DES had different architectural patterns and more favorable clinical outcomes compared to those associated with first-generation DES [26]. This observation is thought to be due to a hypersensitivity reaction after the drug has eluted from the stent, which results in vessel wall weakening and dilation [6]. Other possible risk factors predisposing to CAA development following DES implantation include chronic total occlusion, infarct-related artery implantation, and lesion length exceeding 33 mm or on the left anterior descending artery [27]. Furthermore, patients with CAAs have higher rates of MACE following first-generation DES implantation compared to those without CAAs. While the pathophysiology remains unclear, possible explanations include turbulent blood flow, incomplete stent coverage, or stent malposition following stent placement, all of which lead to increased stent thrombosis and ISR [20]. Despite these findings, the CAAR suggests that, in general, DES can decrease rates of MACE and mortality compared to BMS. Thus, second-generation DES should be the default strategy in patients with significant cardiovascular burdens [1].

Polytetrafluoroethylene (PTFE)-covered stents are another possible treatment option for CAAs but have an increased risk for local thrombosis, ISR, branch artery occlusion, and leakage into the aneurysm sac [8]. These complications are suspected to be related to delayed re-endothelization with polymer-covered stents [1]. In general, PTFE-covered stents can be successful for giant CAAs with diameters of 6-10 mm, but their long-term effectiveness has not been well-studied [1, 28].

### 3.3 Surgical Intervention

Surgical management of CAAs is reserved for symptomatic patients with severe diseases, such as CAD with a high atherosclerotic burden, MI due to embolization despite medical therapy, left coronary artery involvement, giant CAAs, and risk of aneurysmal rupture [7, 8]. Some surgical techniques for CAAs include aneurysm resection, ligation, marsupialization with graft placement, and coronary artery bypass graft [7, 6]. These methods are mainly based on the treatment of atherosclerosis-associated CAAs.

## 4 CLINICAL OUTCOMES

Currently, there is limited data on the prognosis of CAAs compared to CAD. However, isolated CAA without underlying obstructive CAD is associated with improved clinical outcomes [18, 29]. In general, CAA prognosis and disease course are highly dependent on the size, location, etiology, morphology, and the progression of aneurysm, and associated complications [8]. Large size of aneurysm tends to have a poor prognosis as giant CAAs are associated with atherosclerotic obstruction, which can lead to MI, arrhythmias, and sudden cardiac death [6].

## CONCLUSION

CAAs can be uncommon causes of subclinical or clinical presentations of ACS. Although the underlying pathophysiology has not been fully elucidated, atherosclerosis is the main cause of CAAs in adults. Management strategies, which include medical therapy, PCI with stent implantation, and surgical procedures, are focused on reducing the atherosclerotic burden and restoring blood flow, and are similar to those for CAD. However, there are no clear set guidelines for CAA management. Medical therapy is recommended to reduce future thrombotic events and interventions are suggested for severe or high-risk CAAs. The choice of coronary stents (BMS, first-generation DES, second-generation DES, and PTFE-covered stents) requires consideration of CAA morphology, individual patient characteristics, and clinical presentation. While the development of CAAs and higher rates of MACE have been observed following first-generation DES implantation, DES generally confers more protective benefits compared to BMS. Furthermore, second-generation DES placement may be indicated for patients with significant cardiovascular risk factors. Further investigations on the pathophysiology of CAAs can improve our understanding of their role in ACS presentation and assist in the development of standardized treatment recommendations.

## LIST OF ABBREVIATIONS

CAA: Coronary Artery Aneurysms
ACS: Acute Coronary Syndrome
IVUS: Intravascular Ultrasound
MRA: Magnetic Resonance Angiogram
